# Poly[(μ_3_-3,5-di­nitro­benzoato-κ^3^ *O* ^1^:*O* ^1′^:*O* ^3^)(μ_2_-hydroxido-κ^2^ *O*:*O*)copper(II)]

**DOI:** 10.1107/S1600536814004280

**Published:** 2014-02-28

**Authors:** B. Sinha, G. C. Dey, B. Sarkar, A. Roy, Seik Weng Ng, Edward R. T. Tiekink

**Affiliations:** a Department of Chemistry, North Bengal University, Dt. Darjeeling, West Bengal 734 013, India; b Department of Chemistry, University of Malaya, 50603 Kuala Lumpur, Malaysia; c Chemistry Department, Faculty of Science, King Abdulaziz University, PO Box 80203 Jeddah, Saudi Arabia

## Abstract

The title complex, [Cu{μ_3_-O_2_CC_6_H_3_(NO_2_)_2_-3,5}(μ-OH)]_*n*_, features zigzag chains in which successive pairs of Cu^II^ atoms are connected by OH bridges and bidentate carboxyl­ate ligands, leading to six-membered Cu(O)(OCO)Cu rings. The zigzag chains are connected into a three-dimensional architecture by Cu—O(nitro) bonds. The coordination geometry of the Cu^II^ atom is square-pyramidal, with the axial position occupied by the nitro O atom, which forms the longer Cu—O bond. Bifurcated hy­droxy–nitro O—H⋯O hydrogen bonds contribute to the stability of the crystal structure.

## Related literature   

For related Cu^II^ structures featuring Cu(μ_2_-carboxyl­ate)(μ_2_-hydrox­yl)Cu rings, see: You *et al.* (2005 ▶); Chen *et al.* (2006 ▶); Xiao *et al.* (2006 ▶). For additional structural analysis, see: Addison *et al.* (1984 ▶).
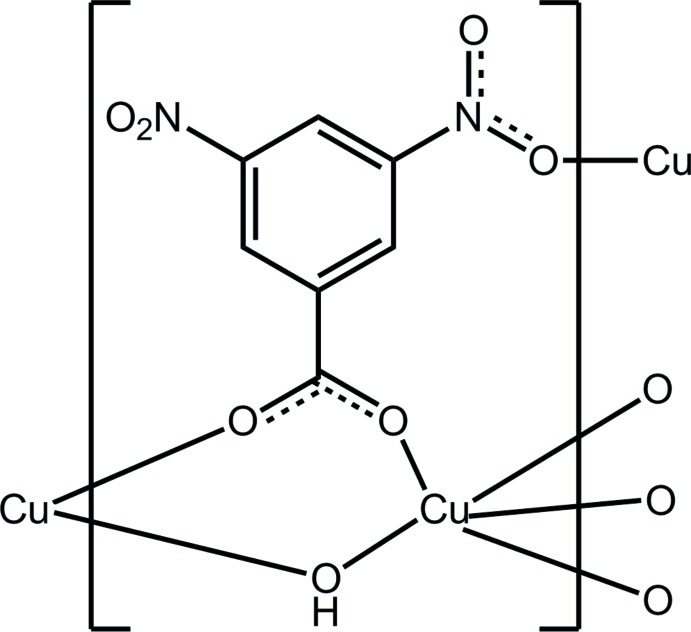


## Experimental   

### 

#### Crystal data  

[Cu(C_7_H_3_N_2_O_6_)(OH)]
*M* *_r_* = 291.66
Orthorhombic, 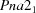

*a* = 7.4665 (2) Å
*b* = 17.7858 (5) Å
*c* = 6.6821 (2) Å
*V* = 887.37 (4) Å^3^
*Z* = 4
Cu *K*α radiation
μ = 3.87 mm^−1^
*T* = 100 K
0.25 × 0.20 × 0.15 mm

#### Data collection  

Agilent SuperNova Dual diffractometer with an Atlas detector
Absorption correction: multi-scan (*CrysAlis PRO*; Agilent, 2011 ▶) *T*_min_ = 0.873, *T*_max_ = 1.000
3156 measured reflections
1324 independent reflections
1318 reflections with *I* > 2σ(*I*)
*R*_int_ = 0.017

#### Refinement  

*R*[*F* ^2^ > 2σ(*F* ^2^)] = 0.027
*wR*(*F* ^2^) = 0.077
*S* = 1.05
1324 reflections
155 parameters
1 restraint
H-atom parameters constrained
Δρ_max_ = 0.45 e Å^−3^
Δρ_min_ = −0.67 e Å^−3^
Absolute structure: Flack (1983 ▶), 360 Friedel pairs
Absolute structure parameter: 0.06 (5)

### 

Data collection: *CrysAlis PRO* (Agilent, 2011 ▶); cell refinement: *CrysAlis PRO*; data reduction: *CrysAlis PRO*; program(s) used to solve structure: *SHELXS97* (Sheldrick, 2008 ▶); program(s) used to refine structure: *SHELXL97* (Sheldrick, 2008 ▶); molecular graphics: *ORTEP-3 for Windows* (Farrugia, 2012 ▶) and *DIAMOND* (Brandenburg, 2006 ▶); software used to prepare material for publication: *publCIF* (Westrip, 2010 ▶).

## Supplementary Material

Crystal structure: contains datablock(s) general, I. DOI: 10.1107/S1600536814004280/hg5386sup1.cif

Structure factors: contains datablock(s) I. DOI: 10.1107/S1600536814004280/hg5386Isup2.hkl

CCDC reference: 988599

Additional supporting information:  crystallographic information; 3D view; checkCIF report

## Figures and Tables

**Table 1:** Selected bond lengths (Å)

Cu—O1	1.9689 (18)
Cu—O7	1.899 (2)
Cu—O2^i^	1.9675 (18)
Cu—O5^ii^	2.5871 (18)
Cu—O7^i^	1.900 (2)

Symmetry codes: (i) 
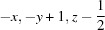
; (ii) 
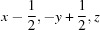
.

**Table 2:** Hydrogen-bond geometry (Å, °)

*D*—H⋯*A*	*D*—H	H⋯*A*	*D*⋯*A*	*D*—H⋯*A*
O7—H1⋯O3^iii^	0.84	2.52	3.190 (3)	137
O7—H1⋯O4^iii^	0.84	2.57	3.271 (2)	142

Symmetry code: (iii) 
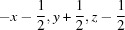
.

**Fig. 1.**
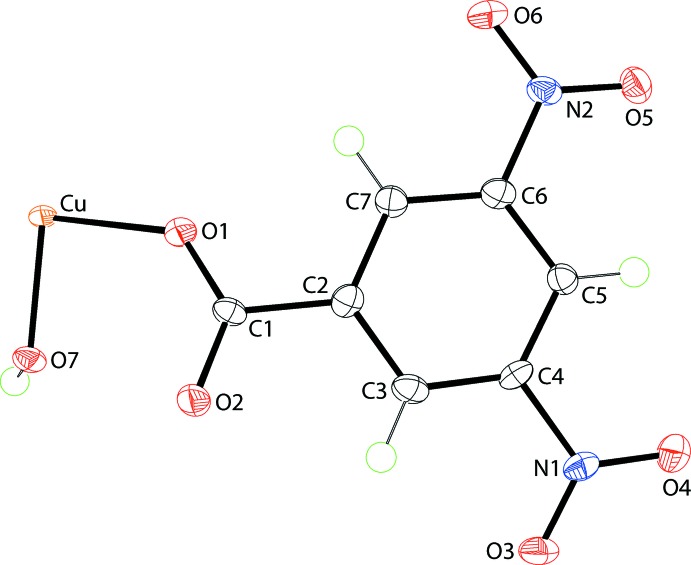
The asymmetric unit of the title compound showing the atom-labelling scheme and displacement ellipsoids at the 70% probability level.

**Fig. 2.**
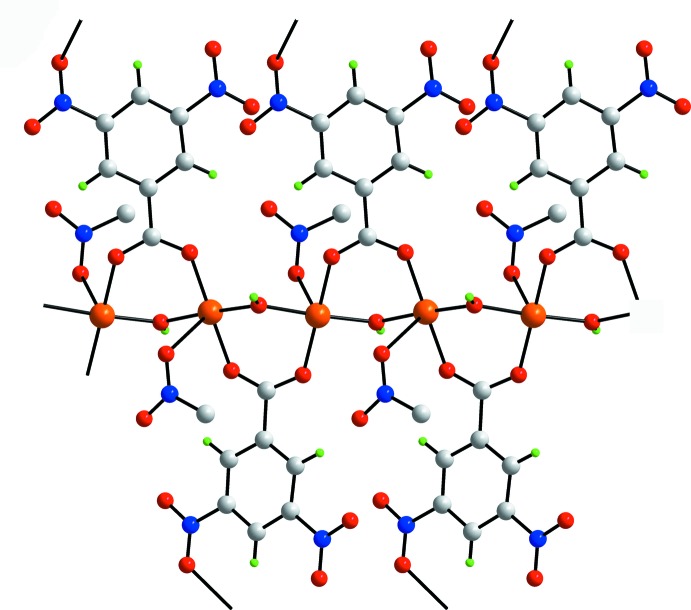
A view approximately normal to the zigzag chain along the c axis in the title compound. The coordinating nitro groups from carboxylates belong to other chains are shown as "O2NC", for reasons of clarity.

**Fig. 3.**
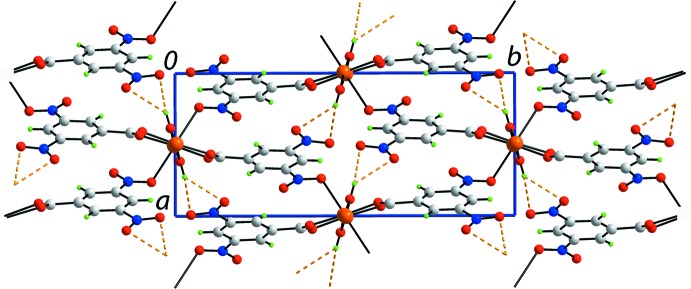
A view of the unit-cell contents of the title compound in projection down the c axis, the axis along which the zigzag chains are propagated. The O—H···O hydrogen bonds are shown as orange dashed lines.

## supplementary crystallographic information

### 2. Structural commentary

The title complex was synthesised employing hydro­thermal methods, and X-ray crystallography revealed it to be three-dimensional. The crystallographic asymmetric unit comprises a Cu^II^ cation, and 3,5-di­nitro­benzoate and hydroxide anions, Fig. 1. Zigzag rows of Cu^II^ ions are aligned along the *c* axis (glide symmetry) with pairs of Cu^II^ ions being bridged by a hydroxide and two O atoms of the carboxyl­ate ligand leading to chains of six-membered rings. Neighbouring chains are linked *via* Cu—O(nitro) bonds, which are longer than the remaining Cu—O bonds, Table 1. The resulting O_5_ donor set defines an axially distorted square pyramidal coordination geometry, with the nitro-O atom in the axial position, as qu­anti­fied by the value of τ = 0.01 which compares to the τ values of 0.0 and 1.0 for ideal square pyramidal and trigonal bipyramidal geometries, respectively (Addison *et al.*, 1984). Additional stability to the architecture is provided by bifurcated O—H···O(nitro) hydrogen bonds, Table 2.

Similar strings of Cu(µ_2_-carboxyl­ate)(µ_2_-hydroxyl)Cu of varying dimensionality have been noted in other Cu^II^ structures (*e.g*. Chen *et al.*, 2006; You *et al.*, 2005; Xiao *et al.*, 2006).

### 5. Synthesis and crystallization

To a pulverised mixture of 3,5-di­nitro­benzoic acid (0.1688 g), Cu(NO_3_)_2_.3H_2_O (0.1932 g) and melamine (0.1002 g), all obtained from commercial sources in AR grade, distilled water (1.5 ml) was added. The mixture was stirred for 30 min to get a suspension. The reaction mixture was then sealed in a 10 ml Teflon-lined stainless steel autoclave and heated at 423 K for 45 h. The autoclave was subjected to natural cooling (for 5 h) to room temperature. The product containing blue crystals suitable for single crystal X-ray diffraction was collected by filtration and washed with adequate distilled water. The initial pH of the suspension was 5 and there was no apparent change in the pH when the reaction was over. The blue product was not formed in the absence of melamine which suggests that melamine acted as a base in this reaction. The product decomposed with explosion with green flashes above 553 K.

### 6. Refinement

The H atoms were geometrically placed (O—H = 0.84 Å and C—H = 0.95 Å) and refined as riding with *U_iso_*(H) = 1.2–1.5*U_eq_*(O, C).

### Crystal data

**Table:**

[Cu(C_7_H_3_N_2_O_6_)(OH)]	*F*(000) = 580
*M**_r_* = 291.66	*D*_x_ = 2.183 Mg m^−^^3^
Orthorhombic, *P**n**a*2_1_	Cu *K*α radiation, λ = 1.54184 Å
Hall symbol: P 2c -2n	Cell parameters from 2719 reflections
*a* = 7.4665 (2) Å	θ = 5.0–74.1°
*b* = 17.7858 (5) Å	µ = 3.87 mm^−^^1^
*c* = 6.6821 (2) Å	*T* = 100 K
*V* = 887.37 (4) Å^3^	Prism, blue
*Z* = 4	0.25 × 0.20 × 0.15 mm

### Data collection

**Table:**

Agilent SuperNova Dual diffractometer with an Atlas detector	1324 independent reflections
Radiation source: SuperNova (Cu) X-ray Source	1318 reflections with *I* > 2σ(*I*)
Mirror monochromator	*R*_int_ = 0.017
Detector resolution: 10.4041 pixels mm^-1^	θ_max_ = 74.3°, θ_min_ = 5.0°
ω scan	*h* = −6→9
Absorption correction: multi-scan (*CrysAlis PRO*; Agilent, 2011)	*k* = −20→21
*T*_min_ = 0.873, *T*_max_ = 1.000	*l* = −7→8
3156 measured reflections	

### Refinement

**Table:**

Refinement on *F*^2^	Hydrogen site location: inferred from neighbouring sites
Least-squares matrix: full	H-atom parameters constrained
*R*[*F*^2^ > 2σ(*F*^2^)] = 0.027	*w* = 1/[σ^2^(*F*_o_^2^) + (0.0617*P*)^2^ + 0.2234*P*] where *P* = (*F*_o_^2^ + 2*F*_c_^2^)/3
*wR*(*F*^2^) = 0.077	(Δ/σ)_max_ = 0.001
*S* = 1.05	Δρ_max_ = 0.45 e Å^−^^3^
1324 reflections	Δρ_min_ = −0.67 e Å^−^^3^
155 parameters	Extinction correction: *SHELXL97* (Sheldrick, 2008), Fc^*^=kFc[1+0.001xFc^2^λ^3^/sin(2θ)]^-1/4^
1 restraint	Extinction coefficient: 0.0078 (6)
Primary atom site location: structure-invariant direct methods	Absolute structure: Flack (1983), 360 Friedel pairs
Secondary atom site location: difference Fourier map	Absolute structure parameter: 0.06 (5)

### Special details

**Table:**

Geometry. All e.s.d.'s (except the e.s.d. in the dihedral angle between two l.s. planes) are estimated using the full covariance matrix. The cell e.s.d.'s are taken into account individually in the estimation of e.s.d.'s in distances, angles and torsion angles; correlations between e.s.d.'s in cell parameters are only used when they are defined by crystal symmetry. An approximate (isotropic) treatment of cell e.s.d.'s is used for estimating e.s.d.'s involving l.s. planes.
Refinement. Refinement of *F*^2^ against ALL reflections. The weighted *R*-factor *wR* and goodness of fit *S* are based on *F*^2^, conventional *R*-factors *R* are based on *F*, with *F* set to zero for negative *F*^2^. The threshold expression of *F*^2^ > σ(*F*^2^) is used only for calculating *R*-factors(gt) *etc*. and is not relevant to the choice of reflections for refinement. *R*-factors based on *F*^2^ are statistically about twice as large as those based on *F*, and *R*- factors based on ALL data will be even larger.

### Fractional atomic coordinates and isotropic or equivalent isotropic displacement parameters (Å^2^)

**Table:**

	*x*	*y*	*z*	*U*_iso_*/*U*_eq_	
Cu	0.00521 (5)	0.502896 (17)	0.50002 (17)	0.00920 (17)	
O1	0.0918 (2)	0.40286 (10)	0.5811 (3)	0.0146 (4)	
O2	0.0673 (3)	0.39533 (9)	0.9179 (3)	0.0136 (4)	
O3	−0.0991 (3)	0.14441 (11)	1.1700 (3)	0.0155 (4)	
O4	−0.0088 (2)	0.04569 (11)	1.0096 (4)	0.0186 (4)	
O5	0.2389 (2)	0.07033 (9)	0.3473 (3)	0.0141 (4)	
O6	0.3041 (2)	0.18099 (10)	0.2336 (3)	0.0187 (4)	
O7	−0.1161 (2)	0.51396 (9)	0.7481 (3)	0.0105 (4)	
H1	−0.2242	0.5270	0.7463	0.016*	
N1	−0.0260 (3)	0.11347 (12)	1.0269 (4)	0.0124 (5)	
N2	0.2439 (3)	0.13903 (11)	0.3632 (3)	0.0119 (4)	
C1	0.0847 (3)	0.36866 (12)	0.7455 (4)	0.0099 (4)	
C2	0.0984 (3)	0.28424 (13)	0.7318 (4)	0.0110 (5)	
C3	0.0397 (4)	0.23927 (14)	0.8891 (4)	0.0121 (5)	
H3	−0.0025	0.2611	1.0100	0.014*	
C4	0.0445 (4)	0.16166 (14)	0.8649 (4)	0.0109 (5)	
C5	0.1101 (4)	0.12656 (13)	0.6959 (4)	0.0108 (5)	
H5	0.1135	0.0734	0.6839	0.013*	
C6	0.1710 (3)	0.17364 (13)	0.5442 (4)	0.0113 (5)	
C7	0.1637 (3)	0.25149 (13)	0.5565 (4)	0.0111 (5)	
H7	0.2024	0.2819	0.4478	0.013*	

### Atomic displacement parameters (Å^2^)

**Table:**

	*U*^11^	*U*^22^	*U*^33^	*U*^12^	*U*^13^	*U*^23^
Cu	0.0143 (3)	0.0092 (2)	0.0042 (3)	0.00052 (10)	0.00062 (13)	0.00047 (15)
O1	0.0228 (10)	0.0118 (8)	0.0092 (9)	0.0025 (6)	0.0030 (7)	0.0015 (7)
O2	0.0210 (10)	0.0118 (8)	0.0081 (9)	0.0024 (7)	−0.0017 (7)	−0.0004 (7)
O3	0.0177 (9)	0.0182 (9)	0.0105 (9)	−0.0005 (8)	0.0047 (7)	−0.0002 (7)
O4	0.0268 (11)	0.0112 (8)	0.0179 (10)	−0.0027 (6)	0.0038 (8)	0.0013 (10)
O5	0.0173 (9)	0.0119 (8)	0.0129 (9)	0.0024 (6)	−0.0015 (7)	−0.0019 (7)
O6	0.0258 (10)	0.0180 (9)	0.0122 (9)	−0.0006 (7)	0.0079 (9)	0.0013 (8)
O7	0.0124 (9)	0.0129 (7)	0.0061 (8)	0.0010 (6)	−0.0013 (8)	−0.0012 (8)
N1	0.0134 (10)	0.0153 (10)	0.0085 (12)	0.0000 (8)	−0.0008 (8)	0.0025 (9)
N2	0.0131 (10)	0.0150 (9)	0.0075 (10)	0.0030 (7)	−0.0005 (8)	−0.0006 (8)
C1	0.0092 (10)	0.0126 (11)	0.0080 (11)	0.0015 (8)	−0.0009 (10)	−0.0015 (10)
C2	0.0111 (10)	0.0121 (11)	0.0099 (12)	0.0004 (9)	−0.0028 (11)	0.0014 (11)
C3	0.0124 (11)	0.0158 (13)	0.0080 (12)	0.0018 (9)	−0.0014 (10)	−0.0014 (10)
C4	0.0116 (11)	0.0130 (12)	0.0081 (12)	−0.0019 (9)	−0.0010 (11)	0.0033 (9)
C5	0.0118 (11)	0.0109 (11)	0.0098 (13)	0.0012 (9)	−0.0010 (9)	−0.0007 (9)
C6	0.0113 (11)	0.0144 (10)	0.0081 (13)	0.0010 (8)	−0.0022 (9)	−0.0003 (8)
C7	0.0121 (11)	0.0117 (10)	0.0095 (12)	−0.0004 (9)	−0.0016 (9)	−0.0009 (8)

### Geometric parameters (Å, º)

**Table:**

Cu—O1	1.9689 (18)	O7—H1	0.8400
Cu—O7	1.899 (2)	N1—C4	1.478 (3)
Cu—O2^i^	1.9675 (18)	N2—C6	1.462 (3)
Cu—O5^ii^	2.5871 (18)	C1—C2	1.508 (3)
Cu—O7^i^	1.900 (2)	C2—C3	1.392 (4)
O1—C1	1.257 (4)	C2—C7	1.396 (4)
O2—C1	1.252 (3)	C3—C4	1.390 (4)
O2—Cu^iii^	1.9675 (18)	C3—H3	0.9500
O3—N1	1.231 (3)	C4—C5	1.380 (4)
O4—N1	1.218 (3)	C5—C6	1.391 (3)
O5—N2	1.227 (3)	C5—H5	0.9500
O6—N2	1.228 (3)	C6—C7	1.388 (3)
O7—Cu^iii^	1.900 (2)	C7—H7	0.9500
			
O7—Cu—O7^i^	176.03 (4)	O2—C1—O1	128.7 (2)
O7—Cu—O2^i^	90.98 (8)	O2—C1—C2	116.1 (2)
O7^i^—Cu—O2^i^	91.02 (9)	O1—C1—C2	115.2 (2)
O7—Cu—O1	90.57 (9)	C3—C2—C7	120.3 (2)
O7^i^—Cu—O1	87.60 (8)	C3—C2—C1	120.3 (2)
O2^i^—Cu—O1	176.81 (9)	C7—C2—C1	119.4 (2)
O7—Cu—O5^ii^	91.71 (7)	C4—C3—C2	118.3 (2)
O7^i^—Cu—O5^ii^	84.60 (8)	C4—C3—H3	120.8
O2^i^—Cu—O5^ii^	98.13 (7)	C2—C3—H3	120.8
O1—Cu—O5^ii^	84.60 (7)	C5—C4—C3	123.6 (2)
C1—O1—Cu	131.60 (17)	C5—C4—N1	117.6 (2)
C1—O2—Cu^iii^	129.32 (16)	C3—C4—N1	118.8 (2)
Cu—O7—Cu^iii^	123.32 (10)	C4—C5—C6	116.1 (2)
Cu—O7—H1	118.3	C4—C5—H5	122.0
Cu^iii^—O7—H1	118.3	C6—C5—H5	122.0
O4—N1—O3	124.3 (2)	C7—C6—C5	123.0 (2)
O4—N1—C4	117.8 (2)	C7—C6—N2	118.9 (2)
O3—N1—C4	117.9 (2)	C5—C6—N2	118.1 (2)
O5—N2—O6	123.7 (2)	C6—C7—C2	118.6 (2)
O5—N2—C6	118.7 (2)	C6—C7—H7	120.7
O6—N2—C6	117.62 (19)	C2—C7—H7	120.7
			
O7—Cu—O1—C1	−8.5 (2)	O4—N1—C4—C5	6.0 (3)
O7^i^—Cu—O1—C1	175.0 (2)	O3—N1—C4—C5	−173.7 (2)
O2^i^—Cu—O7—Cu^iii^	−127.01 (11)	O4—N1—C4—C3	−174.7 (2)
O1—Cu—O7—Cu^iii^	50.20 (11)	O3—N1—C4—C3	5.7 (4)
Cu^iii^—O2—C1—O1	14.1 (4)	C3—C4—C5—C6	−0.9 (4)
Cu^iii^—O2—C1—C2	−165.63 (15)	N1—C4—C5—C6	178.3 (2)
Cu—O1—C1—O2	−22.7 (4)	C4—C5—C6—C7	−1.5 (3)
Cu—O1—C1—C2	156.98 (16)	C4—C5—C6—N2	179.2 (2)
O2—C1—C2—C3	19.2 (3)	O5—N2—C6—C7	−175.1 (2)
O1—C1—C2—C3	−160.6 (2)	O6—N2—C6—C7	3.6 (3)
O2—C1—C2—C7	−163.4 (2)	O5—N2—C6—C5	4.2 (3)
O1—C1—C2—C7	16.8 (3)	O6—N2—C6—C5	−177.0 (2)
C7—C2—C3—C4	−1.3 (4)	C5—C6—C7—C2	2.4 (3)
C1—C2—C3—C4	176.0 (2)	N2—C6—C7—C2	−178.3 (2)
C2—C3—C4—C5	2.3 (4)	C3—C2—C7—C6	−0.9 (3)
C2—C3—C4—N1	−176.9 (2)	C1—C2—C7—C6	−178.3 (2)

Symmetry codes: (i) −*x*, −*y*+1, *z*−1/2; (ii) *x*−1/2, −*y*+1/2, *z*; (iii) −*x*, −*y*+1, *z*+1/2.

### Hydrogen-bond geometry (Å, º)

**Table:**

*D*—H···*A*	*D*—H	H···*A*	*D*···*A*	*D*—H···*A*
O7—H1···O3^iv^	0.84	2.52	3.190 (3)	137
O7—H1···O4^iv^	0.84	2.57	3.271 (2)	142

Symmetry code: (iv) −*x*−1/2, *y*+1/2, *z*−1/2.
